# Supplementary material for: Molecular Characterization of the Extracellular Domain of Human Junctional Adhesion Proteins
Source: Int J Mol Sci. 2021 Mar 27;22(7):3482. doi: 10.3390/ijms22073482 (PMC8037251; doi:10.3390/ijms22073482)
Supplement: Supplementary file 1 [file ijms-22-03482-s001.pdf]

**References**


1. Balda, M.S.; Matter, K. Tight junctions at a glance. J Cell Sci 2008, 121, 3677-3682, doi:10.1242/jcs.023887.
2. Balda, M.S.; Matter, K. Tight junctions and the regulation of gene expression. Biochim Biophys Acta 2009, 1788, 761-767, doi:10.1016/j.bbamem.2008.11.024.
3. Cheng, P.; Yao, J.; Wang, C.; Zhang, L.; Kong, W. Molecular and cellular mechanisms of tight junction dysfunction in the irritable bowel syndrome. Mol Med Rep 2015, 12, 3257-3264, doi:10.3892/mmr.2015.3808.
4. Lee, D.B.; Huang, E.; Ward, H.J. Tight junction biology and kidney dysfunction. Am J Physiol Renal Physiol 2006, 290, F20-34, doi:10.1152/ajprenal.00052.2005.
5. Zhang, J.B.; Du, X.G.; Zhang, H.; Li, M.L.; Xiao, G.; Wu, J.; Gan, H. Breakdown of the gut barrier in patients with multiple organ dysfunction syndrome is attenuated by continuous blood purification: effects on tight junction structural proteins. Int J Artif Organs 2010, 33, 5-14.
6. Zheng, G.; Victor Fon, G.; Meixner, W.; Creekmore, A.; Zong, Y.; M, K.D.; Colacino, J.; Dedhia, P.H.; Hong, S.; Wiley, J.W. Chronic stress and intestinal barrier dysfunction: Glucocorticoid receptor and transcription repressor HES1 regulate tight junction protein Claudin-1 promoter. Sci Rep 2017, 7, 4502, doi:10.1038/s41598-017-04755-w.
7. Angulo-Urarte, A.; van der Wal, T.; Huveneers, S. Cell-cell junctions as sensors and transducers of mechanical forces. Biochim Biophys Acta Biomembr 2020, 1862, 183316, doi:10.1016/j.bbamem.2020.183316.
8. Citi, S. The mechanobiology of tight junctions. Biophys Rev 2019, 11, 783-793, doi:10.1007/s12551-019-00582-7.
9. Greene, C.; Campbell, M. Tight junction modulation of the blood brain barrier: CNS delivery of small molecules. Tissue Barriers 2016, 4, e1138017, doi:10.1080/21688370.2015.1138017.
10. Greene, C.; Hanley, N.; Campbell, M. Claudin-5: gatekeeper of neurological function. Fluids Barriers CNS 2019, 16, 3, doi:10.1186/s12987-019-0123-z.
11. Otani, T.; Furuse, M. Tight Junction Structure and Function Revisited. Trends Cell Biol 2020, 30, 805-817, doi:10.1016/j.tcb.2020.08.004.
12. Kummer, D.; Ebnet, K. Junctional Adhesion Molecules (JAMs): The JAM-Integrin Connection. Cells 2018, 7, doi:10.3390/cells7040025.
13. Steinbacher, T.; Kummer, D.; Ebnet, K. Junctional adhesion molecule-A: functional diversity through molecular promiscuity. Cell Mol Life Sci 2018, 75, 1393-1409, doi:10.1007/s00018-017-2729-0.
14. Hirabayashi, S.; Tajima, M.; Yao, I.; Nishimura, W.; Mori, H.; Hata, Y. JAM4, a junctional cell adhesion molecule interacting with a tight junction protein, MAGI-1. Mol Cell Biol 2003, 23, 4267-4282, doi:10.1128/mcb.23.12.4267-4282.2003.
15. Nagamatsu, G.; Ohmura, M.; Mizukami, T.; Hamaguchi, I.; Hirabayashi, S.; Yoshida, S.; Hata, Y.; Suda, T.; Ohbo, K. A CTX family cell adhesion molecule, JAM4, is expressed in stem cell and progenitor cell populations of both male germ cell and hematopoietic cell lineages. Mol Cell Biol 2006, 26, 8498-8506, doi:10.1128/MCB.01502-06.
16. Weber, C.; Fraemohs, L.; Dejana, E. The role of junctional adhesion molecules in vascular inflammation. Nat Rev Immunol 2007, 7, 467-477, doi:10.1038/nri2096.
17. Mandell, K.J.; McCall, I.C.; Parkos, C.A. Involvement of the junctional adhesion molecule-1 (JAM1) homodimer interface in regulation of epithelial barrier function. J Biol Chem 2004, 279, 16254-16262, doi:10.1074/jbc.M309483200.
18. Cera, M.R.; Del Prete, A.; Vecchi, A.; Corada, M.; Martin-Padura, I.; Motoike, T.; Tonetti, P.; Bazzoni, G.; Vermi, W.; Gentili, F.; et al. Increased DC trafficking to lymph nodes and contact hypersensitivity in junctional adhesion molecule-A-deficient mice. J Clin Invest 2004, 114, 729-738, doi:10.1172/JCI21231.

19. Ebnet, K. Junctional Adhesion Molecules (JAMs): Cell Adhesion Receptors With Pleiotropic Functions in Cell Physiology and Development. Physiol Rev 2017, 97, 1529-1554, doi:10.1152/physrev.00004.2017.
20. Aceto, N.; Toner, M.; Maheswaran, S.; Haber, D.A. En Route to Metastasis: Circulating Tumor Cell Clusters and Epithelial-to-Mesenchymal Transition. Trends Cancer 2015, 1, 44-52, doi:10.1016/j.trecan.2015.07.006.
21. Tian, Y.; Tian, Y.; Zhang, W.; Wei, F.; Yang, J.; Luo, X.; Zhou, T.; Hou, B.; Qian, S.; Deng, X.; et al. Junctional adhesion molecule-A, an epithelial-mesenchymal transition inducer, correlates with metastasis and poor prognosis in human nasopharyngeal cancer. Carcinogenesis 2015, 36, 41-48, doi:10.1093/carcin/bgu230.
22. Prota, A.E.; Campbell, J.A.; Schelling, P.; Forrest, J.C.; Watson, M.J.; Peters, T.R.; Aurrand-Lions, M.; Imhof, B.A.; Dermody, T.S.; Stehle, T. Crystal structure of human junctional adhesion molecule 1: implications for reovirus binding. Proc Natl Acad Sci U S A 2003, 100, 5366-5371, doi:10.1073/pnas.0937718100.
23. Tate, C.G. Overexpression of mammalian integral membrane proteins for structural studies. FEBS Lett 2001, 504, 94-98, doi:10.1016/s0014-5793(01)02711-9.
24. Roosild, T.P.; Greenwald, J.; Vega, M.; Castronovo, S.; Riek, R.; Choe, S. NMR structure of Mistic, a membrane-integrating protein for membrane protein expression. Science 2005, 307, 1317-1321, doi:10.1126/science.1106392.
25. Bill, R.M.; Goddard, A.D.; Rothnie, A.J. Recombinant Membrane Protein Methods. Methods 2018, 147, 1-2, doi:10.1016/j.ymeth.2018.08.007.
26. van Roy, F.; Berx, G. The cell-cell adhesion molecule E-cadherin. Cell Mol Life Sci 2008, 65, 3756-3788, doi:10.1007/s00018-008-8281-1.
27. Vendome, J.; Felsovalyi, K.; Song, H.; Yang, Z.; Jin, X.; Brasch, J.; Harrison, O.J.; Ahlsen, G.; Bahna, F.; Kaczynska, A.; et al. Structural and energetic determinants of adhesive binding specificity in type I cadherins. Proc Natl Acad Sci U S A 2014, 111, E4175-4184, doi:10.1073/pnas.1416737111.
28. Katsamba, P.; Carroll, K.; Ahlsen, G.; Bahna, F.; Vendome, J.; Posy, S.; Rajebhosale, M.; Price, S.; Jessell, T.M.; Ben-Shaul, A.; et al. Linking molecular affinity and cellular specificity in cadherin-mediated adhesion. Proc Natl Acad Sci U S A 2009, 106, 11594-11599, doi:10.1073/pnas.0905349106.
29. Gawdi, R.; Emmady, P.D. Physiology, Blood Brain Barrier. In StatPearls; Treasure Island (FL), 2021.
30. Alvarez, F.J.; Orelle, C.; Huang, Y.; Bajaj, R.; Everly, R.M.; Klug, C.S.; Davidson, A.L. Full engagement of liganded maltose-binding protein stabilizes a semi-open ATP-binding cassette dimer in the maltose transporter. Mol Microbiol 2015, 98, 878-894, doi:10.1111/mmi.13165.
31. Smyth, D.R.; Mrozkiewicz, M.K.; McGrath, W.J.; Listwan, P.; Kobe, B. Crystal structures of fusion proteins with large-affinity tags. Protein Sci 2003, 12, 1313-1322, doi:10.1110/ps.0243403.
32. Lobstein, J.; Emrich, C.A.; Jeans, C.; Faulkner, M.; Riggs, P.; Berkmen, M. SHuffle, a novel Escherichia coli protein expression strain capable of correctly folding disulfide bonded proteins in its cytoplasm. Microb Cell Fact 2012, 11, 56, doi:10.1186/1475-2859-11-56.
33. Parisini, E.; Higgins, J.M.; Liu, J.H.; Brenner, M.B.; Wang, J.H. The crystal structure of human E-cadherin domains 1 and 2, and comparison with other cadherins in the context of adhesion mechanism. J Mol Biol 2007, 373, 401-411, doi:10.1016/j.jmb.2007.08.011.
34. Zhang, F.; Wei, Q.; Tong, H.; Xu, D.; Wang, W.; Ran, T. Crystal structure of MBP-PigG fusion protein and the essential function of PigG in the prodigiosin biosynthetic pathway in Serratia marcescens FS14. Int J Biol Macromol 2017, 99, 394-400, doi:10.1016/j.ijbiomac.2017.02.088.

35. Balan, A.; de Souza, C.S.; Moutran, A.; Ferreira, R.C.; Franco, C.S.; Ramos, C.H.; de Souza Ferreira, L.C. Purification and in vitro characterization of the maltose-binding protein of the plant pathogen Xanthomonas citri. Protein Expr Purif 2005, 43, 103-110, doi:10.1016/j.pep.2005.03.018.
36. Quiocho, F.A.; Spurlino, J.C.; Rodseth, L.E. Extensive features of tight oligosaccharide binding revealed in high-resolution structures of the maltodextrin transport/chemosensory receptor. Structure 1997, 5, 997-1015, doi:10.1016/s0969-2126(97)00253-0.
37. Pattnaik, P. Surface plasmon resonance: applications in understanding receptor-ligand interaction. Appl Biochem Biotechnol 2005, 126, 79-92, doi:10.1385/abab:126:2:079.
38. Tang, Y.; Zeng, X.; Liang, J. Surface Plasmon Resonance: An Introduction to a Surface Spectroscopy Technique. J Chem Educ 2010, 87, 742-746, doi:10.1021/ed100186y.
39. Gliki, G.; Ebnet, K.; Aurrand-Lions, M.; Imhof, B.A.; Adams, R.H. Spermatid differentiation requires the assembly of a cell polarity complex downstream of junctional adhesion molecule-C. Nature 2004, 431, 320-324, doi:10.1038/nature02877.
40. Reglero-Real, N.; Colom, B.; Bodkin, J.V.; Nourshargh, S. Endothelial Cell Junctional Adhesion Molecules: Role and Regulation of Expression in Inflammation. Arterioscler Thromb Vasc Biol 2016, 36, 2048-2057, doi:10.1161/ATVBAHA.116.307610.
41. Del Vecchio, G.; Tscheik, C.; Tenz, K.; Helms, H.C.; Winkler, L.; Blasig, R.; Blasig, I.E. Sodium caprate transiently opens claudin-5-containing barriers at tight junctions of epithelial and endothelial cells. Mol Pharm 2012, 9, 2523-2533, doi:10.1021/mp3001414.
42. Sun, T.; Hevner, R.F. Growth and folding of the mammalian cerebral cortex: from molecules to malformations. Nat Rev Neurosci 2014, 15, 217-232, doi:10.1038/nrn3707.
43. Ebnet, K.; Suzuki, A.; Ohno, S.; Vestweber, D. Junctional adhesion molecules (JAMs): more molecules with dual functions? J Cell Sci 2004, 117, 19-29, doi:10.1242/jcs.00930.
44. Campbell, H.K.; Maiers, J.L.; DeMali, K.A. Interplay between tight junctions & adherens junctions. Exp Cell Res 2017, 358, 39-44, doi:10.1016/j.yexcr.2017.03.061.
45. Pettersen, E.F.; Goddard, T.D.; Huang, C.C.; Couch, G.S.; Greenblatt, D.M.; Meng, E.C.; Ferrin, T.E. UCSF Chimera--a visualization system for exploratory research and analysis. J Comput Chem 2004, 25, 1605-1612, doi:10.1002/jcc.20084.
46. Merril, C.R. Gel-staining techniques. Methods Enzymol 1990, 182, 477-488, doi:10.1016/0076-6879(90)82038-4.
47. Fischer, M.J.E. Amine Coupling Through EDC/NHS: A Practical Approach. Humana Press: 2010; pp. 55-73.

## Supplementary Information

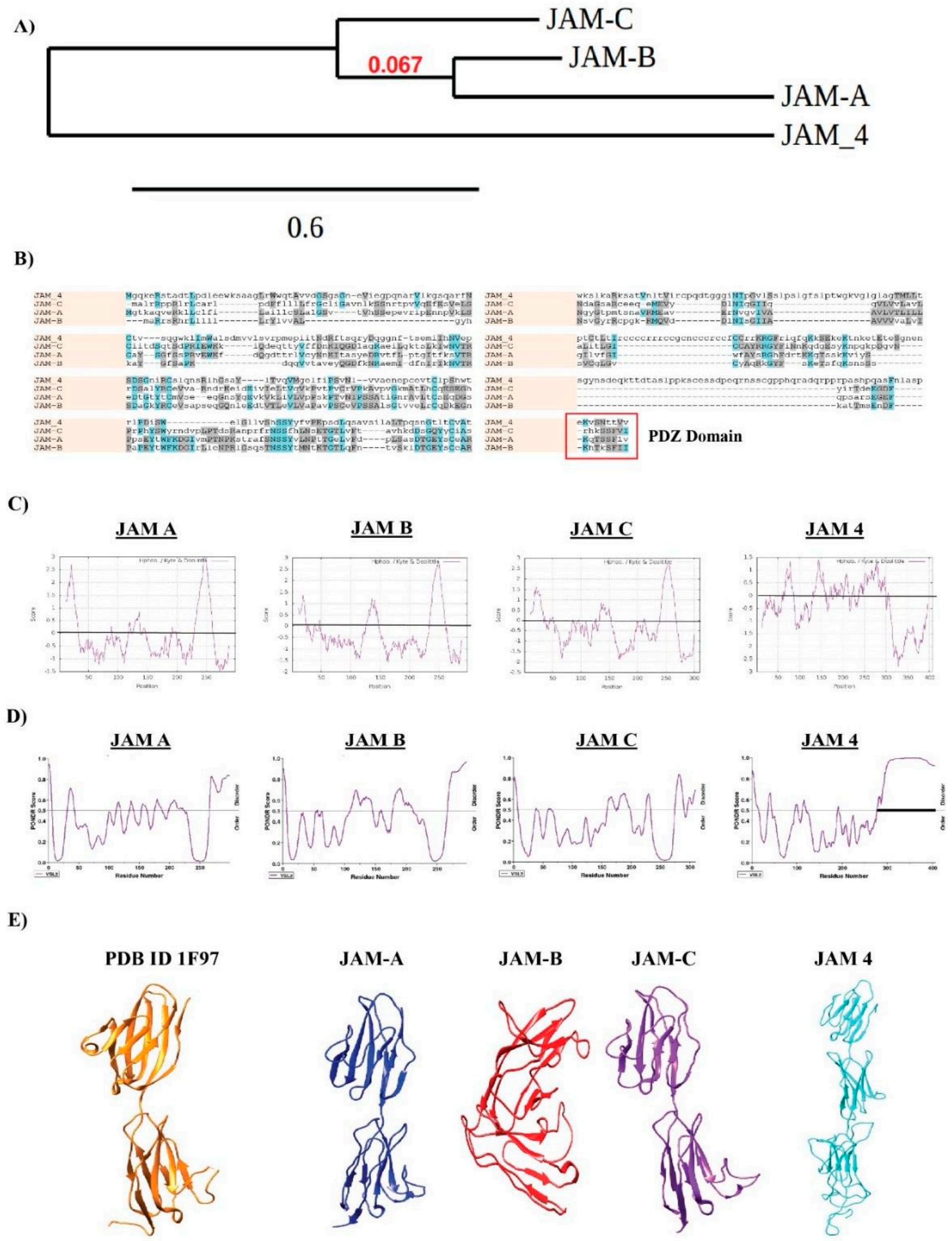


**Supplementary Figure 1. Conserved structures of JAM proteins.** A) Phylogenetic tree of JAM proteins. B) Amino acid sequence alignment of JAM proteins. C) Hydropathy plots of JAMs. D) Order and disorder regions of JAMs. E) Models of JAM proteins. See Materials and Methods.

**Supplementary Figure 2.** pET28-MBP (Kanamycin resistance)

```
        10         20         30         40         50         60
MGKIEEGKLV IWINGDKGYN GLAEVGKKFE KDTGIKVTVE HPDKLEEKFP QVAATGDGPD

        70         80         90        100        110        120
IIFWAHDRFG GYAQSGLLAE ITPDKAFQDK LYPFTWDAVR YNGKLIAYPI AVEALSLIYN

       130        140        150        160        170        180
KDLLPNPPKT WEEIPALDKE LKAKGKSALM FNLQEPYFTW PLIAADGGYA FKYENGKYDI

       190        200        210        220        230        240
KDVGVDNAGA KAGLTFLVDL IKNKHMNADT DYSIAEAAFN KGETAMTING PWAWSNIDTS

       250        260        270        280        290        300
KVNYGVTVLP TFKGQPSKPF VGVLSAGINA ASPNKELAKE FLENYLLTDE GLEAVNKDKP

       310        320        330        340        350        360
LGAVALKSYE EELAKDPRIA ATMENAQKGE IMPNIPQMSA FWYAVRTAVI NAASGRQTVD

       370
EALKDAQTNA AAHM -INSERT-LEHHHHHH
```

**Number of amino acids:** 374. **Molecular weight:** 40992.61

Sites for NdeI (**HM** amino acids) and XhoI (LE amino acids) are present in the plasmid.

Example **pET28-MBP-JAM-A-6xHIS**

```
        10         20         30         40         50         60
MGKIEEGKLV IWINGDKGYN GLAEVGKKFE KDTGIKVTVE HPDKLEEKFP QVAATGDGPD

        70         80         90        100        110        120
IIFWAHDRFG GYAQSGLLAE ITPDKAFQDK LYPFTWDAVR YNGKLIAYPI AVEALSLIYN

       130        140        150        160        170        180
KDLLPNPPKT WEEIPALDKE LKAKGKSALM FNLQEPYFTW PLIAADGGYA FKYENGKYDI

       190        200        210        220        230        240
KDVGVDNAGA KAGLTFLVDL IKNKHMNADT DYSIAEAAFN KGETAMTING PWAWSNIDTS

       250        260        270        280        290        300
KVNYGVTVLP TFKGQPSKPF VGVLSAGINA ASPNKELAKE FLENYLLTDE GLEAVNKDKP

       310        320        330        340        350        360
LGAVALKSYE EELAKDPRIA ATMENAQKGE IMPNIPQMSA FWYAVRTAVI NAASGRQTVD

       370        380        390        400        410        420
EALKDAQTNA AAHMGSGSGS VTVHSSEPEV RIPENNPVKL SCAYSGFSSP RVEWKFDQGD

       430        440        450        460        470        480
TTRLVCYNNK ITASYEDRVT FLPTGITFKS VTREDTGTYT CMVSEEGGNS YGEVKVKLIV

       490        500        510        520        530        540
LVPPSKPTVN IPSSATIGNR AVLTCSEQDG SPPSEYTWFK DGIVMPTNPK STRAFSNSSY

       550        560        570        580
VLNPTTGELV FDPLSASDTG EYSCEARNGY GTPMTSNAVR LEHHHHHH
```

**Number of amino acids:** 588. **Molecular weight:** 64250.35

**Supplementary Figure 3.** **<u>gBlock sequences</u>** (IDT DNA Technologies, Codon Optimized for E. coli K-12)

- JAM-A

CTCGAGGGAAGTGGAAGCGGAAGTGTGACTGTTCATAGCAGTGAGCCCGAGGTGCGCATTCCGG
AGAACAATCCAGTGAAATTGAGTTGTGCGTACAGTGGTTTTAGTTCACCACGTGTAGAATGGAA
GTTTGACCAGGGGGATACTACACGCCTTGTGTGTTATAATAATAAAATCACCGCTAGCTACGAG
GACCGTGTCACTTTTTTACCAACAGGTATTACGTTCAAGTCCGTGACCCGCGAGGACACAGGCA
CGTATACATGCATGGTATCGGAAGAGGGCGGGAACTCGTACGGGGAGGTTAAGGTTAAATTGAT
TGTATTAGTCCCACCCTCTAAACCCACAGTGAACATCCCTAGTTCCGCAACAATCGGCAATCGT
GCCGTTTTAACTTGCTCAGAACAAGATGGTTCACCACCCTCAGAATACACATGGTTTAAGGACG
GTATCGTTATGCCTACCAATCCAAAGTCCACCCGTGCATTCAGCAACTCCTCTTATGTGCTTAA
TCCCACTACCGGTGAATTAGTGTTCGACCCCCTGTCCGCTAGTGATACAGGAGAATACTCGTGC
GAAGCCCGTAACGGGTATGGTACACCGATGACTTCCAATGCTGTCCGTATGGAAGCCGTCGAGC
GCAACGTCGGAGTCATCGTAGCGGCGGTGTTAGTCACGTTAATTCTGCTGGGCATTTTGGTGTT
CGGAATTTGGTTTGCATACTCGCGTGGCCATTTCGATCGCACCAAAAAGGGCACTAGTTCCAAG
AAAGTTATTTATAGTCAGCCATCTGCTCGCTCGGAGGGAGAGTTTAAGCAGACATCGTCTTTTC
TTGTTCTCGAG

- JAM-B

CTCGAGGGAAGTGGAAGCGGAAGTTTTAGCGCGCCAAAAGACCAACAGGTTGTGACAGCTGTGG
AATACCAGGAAGCCATTTTAGCGTGCAAGACTCCTAAGAAGACGGTATCGTCGCGTCTTGAATG
GAAAAAGTTGGGGCGCTCGGTTTCCTTTGTGTACTATCAACAAACCCTGCAGGGTGACTTTAAG
AACCGCGCCGAGATGATCGACTTTAATATTCGCATCAAGAACGTCACTCGTTCTGACGCAGGCA
AGTACCGTTGCGAAGTAAGTGCGCCCTCGGAGCAGGGACAAAACTTAGAAGAAGACACAGTCAC
CTTGGAAGTTTTGGTGGCACCAGCAGTCCCCTCATGTGAAGTCCCTTCGTCTGCACTGAGCGGC
ACAGTTGTCGAATTGCGCTGCCAAGATAAAGAGGGAAATCCCGCCCCCGAGTACACATGGTTTA
AGGACGGGATCCGTCTTCTTGAGAATCCCCGTTTGGGGTCTCAATCTACGAATAGTTCATATAC
CATGAACACCAAAACTGGCACGCTGCAGTTTAATACAGTTTCAAAGTTAGACACGGGCGAGTAC
TCGTGCGAAGCTCGCAACTCGGTGGGCTATCGCCGCTGTCCTGGTAAGCGTATGCAAGTCGACG
ACTTGAATATTTCAGGAATCATCGCTGCCGTGGTTGTTGTTGCCCTGGTTATTAGTGTGTGTGG
GCTTGGAGTATGTTATGCCCAACGCAAAGGTTACTTTTCGAAGGAAACATCTTTCCAGAAATCG
AACTCCTCTTCGAAGGCGACTACCATGTCAGAGAATGATTTCAAACATACTAAATCATTTATCA
TTCTCGAG

- JAM-C

CTCGAGGGAAGTGGAAGCGGAAGTGCCGTTAATCTGAAGTCGAGTAACCGTACTCCAGTGGTTC
AAGAGTTCGAGAGTGTAGAGCTTTCATGCATCATCACCGACAGTCAAACTTCGGACCCACGCAT
CGAATGGAAAAAGATCCAAGACGAGCAGACTACGTACGTGTTTTTCGACAACAAAATCCAGGGC
GATTTAGCGGGACGTGCTGAGATCTTAGGGAAAACCAGCCTGAAAATCTGGAACGTAACCCGTC
GCGACAGTGCCTTATATCGTTGCGAGGTTGTGGCTCGCAATGATCGCAAGGAGATCGATGAGAT
CGTAATCGAGCTGACCGTACAAGTCAAACCAGTGACCCCGGTCTGTCGCGTGCCAAAGGCTGTC
CCTGTTGGTAAGATGGCTACTCTTCATTGCCAGGAATCAGAAGGGCACCCTCGCCCCCATTATT
CATGGTACCGCAATGACGTTCCATTGCCCACGGACAGCCGTGCCAACCCACGTTTTCGCAATAG
TTCATTTCACTTGAACTCCGAGACAGGAACATTGGTTTTCACCGCCGTTCACAAAGATGATTCT
GGTCAATACTATTGTATTGCATCTAACGACGCGGGTTCTGCGCGTTGCGAGGAACAAGAAATGG
AAGTCTACGACTTGAACATCGGTGGGATCATCGGTGGGGTGTTAGTGGTTCTTGCCGTGCTGGC

CCTTATCACACTGGGCATCTGTTGTGCGTACCGCCGTGGCTATTTCATCAATAACAAGCAGGAC
GGCGAATCCTACAAGAACCCTGGAAAACCGGATGGAGTAAATTATATCCGCACGGATGAAGAGG
GGGATTTTCGTCACAAATCGAGTTTCGTTATTCTCGAG

- JAM4

GGCTCAGGCAGTGGCAATGAAGTCATCGAGGGACCACAAAATGCTCGTGTGCTGAAGGGAAGTC
AGGCCCGTTTTAATTGCACAGTAAGCCAGGGATGGAAGTTGATTATGTGGGCCCTTTCCGATAT
GGTCGTTTTAAGTGTGCGCCCCATGGAACCAATTATTACAAATGATCGCTTTACCAGTCAACGT
TATGACCAGGGTGGCAATTTCACCTCTGAAATGATTATTCACAATGTAGAACCTAGTGACTCAG
GCAACATTCGCTGTTCGCTTCAAAATAGTCGCTTGCACGGTTCAGCGTATCTGACTGTACAAGT
TATGGGAGAATTGTTTATCCCATCAGTAAACCTTGTGGTAGCTGAAAACGAACCTTGCGAGGTA
ACGTGCTTGCCATCACATTGGACTCGCCTTCCCGACATCAGTTGGGAACTGGGCTTACTTGTCT
CCCATAGTTCATATTACTTCGTGCCCGAACCCAGCGACCTGCAGTCGGCTGTCTCAATCTTGGC
ACTGACCCCTCAGTCGAATGGGACCCTGACATGTGTTGCAACATGGAAATCCTTAAAAGCACGC
AAATCGGCTACGGTTAACCTTACCGTTATCCGCTGTCCTCAGGATACCGGTGGTGGCATTAATA
TTCCCGGTGTGTTATCGTCGCTTCCCTCCTTGGGCTTTTCTCTTCCTACATGGGGAAAGGTGGG
ACTGGGTTTAGCCGGGACTATGTTGCTTACACCGACATGCACGTTAACCATCCGTTGTTGCTGT
TGTCGTCGTCGCTGCTGTGGATGCAATTGCTGTTGCCGTTGTTGCTTCTGCTGTCGCCGTAAAC
GCGGATTTCGCATCCAATTTCAAAAGAAGTCCGAGAAAGAAAAGACAAACAAAGAAACGGAAAC
CGAGTCAGGTAATGAGAACTCAGGGTATAACAGTGATGAGCAAAAGACAACAGATACTGCTTCC
TTACCACCAAAATCTTGTGAGTCCAGCGATCCTGAACAACGCAATTCCTCCTGTGGCCGCCCC
ACCAACGCGCAGATCAACGCCCTCCTCGCCCCGCCTCGCATCCCCAAGCGTCTTTCAACCTTGC
GTCCCCTGAAAAAGTATCCAATACAACCGTCGTC

- E-CAD

TAATACGACTCACTATAGGGCatatgGTGATTCCACCCATCAGTTGCCCGGAAAATGAGAAAGG
GCCTTTCCCAAAAAATCTGGTTCAGATCAAAAGTAACAAGGACAAGGAGGGTAAGGTGTTTTAT
TCTATCACGGGTCAGGGTGCCGACACGCCACCCGTTGGTGTGTTCATCATTGAGCGCGAGACGG
GATGGCTGAAGGTTACTGAACCTCTTGATCGTGAGCGTATCGCGACCTATACGTTGTTTTCACA
TGCTGTAAGTTCAAATGGGAATGCGGTAGAGGACCCGATGGAGATTTTGATTACTGTAACGGAC
CAGAATGACAATAAACCAGAGTTTACGCAAGAGGTATTTAAGGGTTCGGTAATGGAGGGAGCTT
TACCTGGCACTTCAGTTATGGAAGTTACCGCGACCGACGCCGACGACGACGTCAACACCTACAA
TGCTGCCATTGCTTATACAATTCTGTCTCAGGACCCAGAGTTGCCAGACAAGAACATGTTCACA
ATCAACCGTAATACGGGTGTTATTTCAGTTGTCACGACAGGTCTTGACCGTGAATCCTTTCCTA
CCTACACGCTTGTTGTGCAGGCTGCAGATCTGCAAGGAGAAGGGTTAAGCACTACGGCGACAGC
CGTGATTACGGTGACCGACctcgagCCACCGCTGAGCAATAACTA

**Supplementary Figure 4. Growth curve**

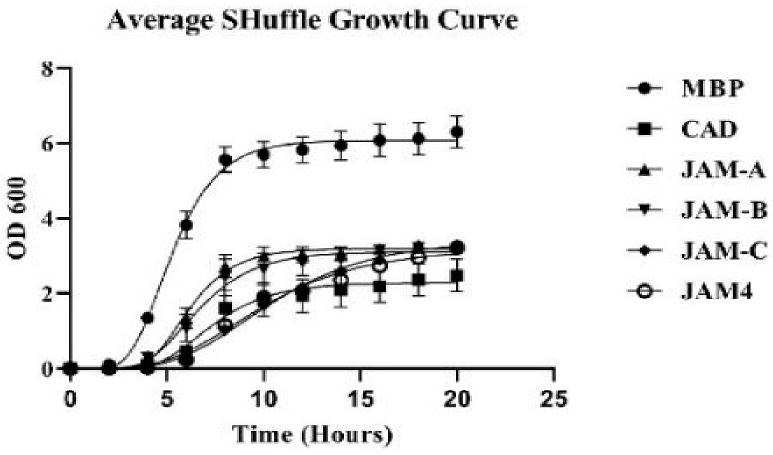


Cells are grown in LB from a 1:1000 dilution of overnight culture of SHuffle cells. Cell growth is monitored ($OD_{600}$) every hour for 20 hours.

**Supplementary Figure 5. Proteins thermal stability by Circular Dichroism,**

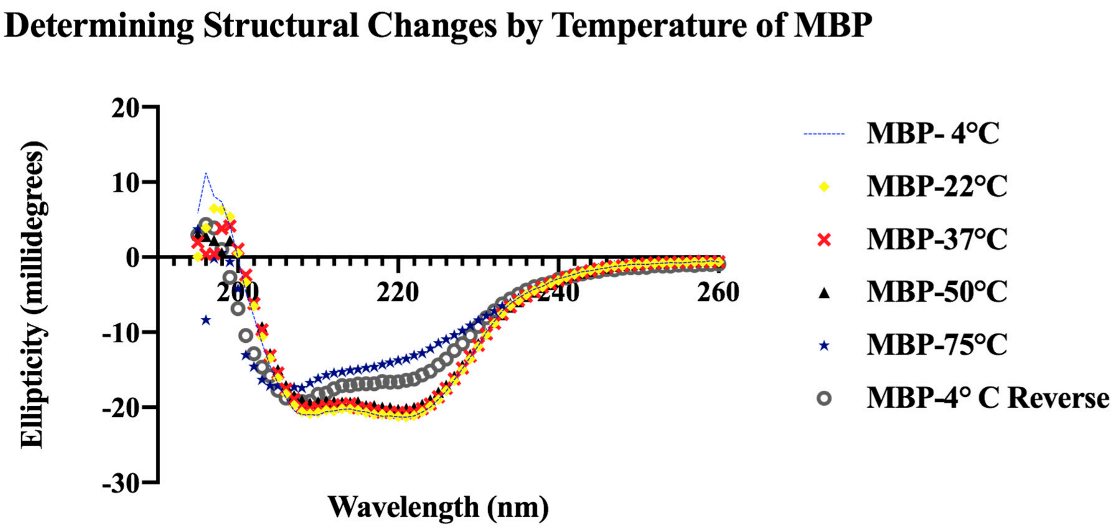

## Determining Structural Changes by Temperature of CAD

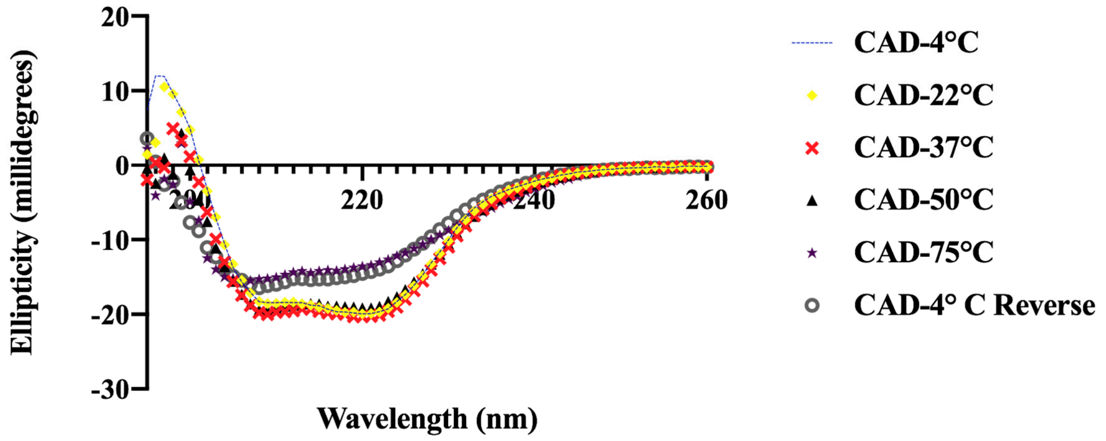

**Determining Structural Changes by Temperature of JAM-A**

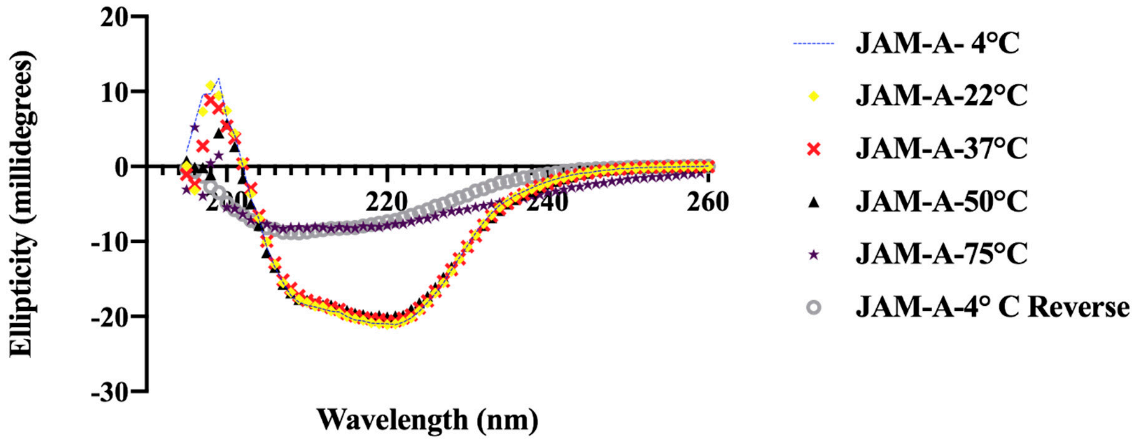


**Determining Structural Changes by Temperature of JAM-B**

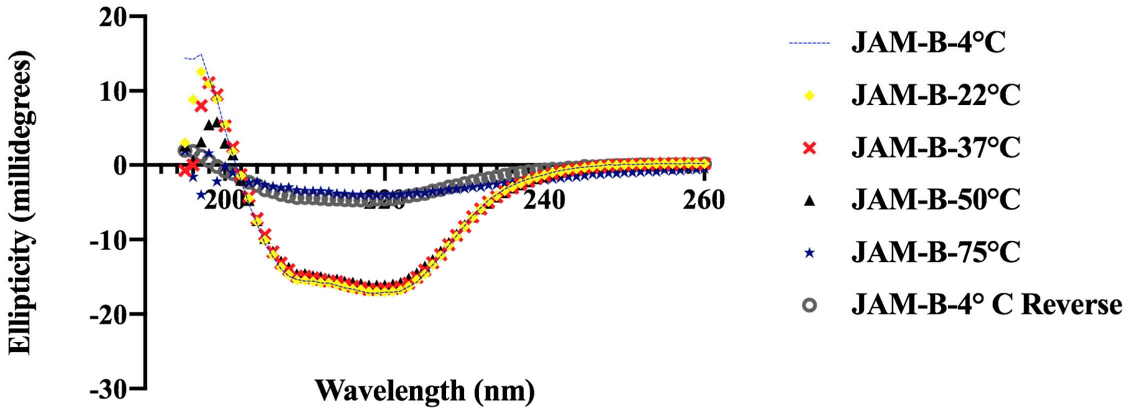

**Determining Structural Changes by Temperature of JAM-C**

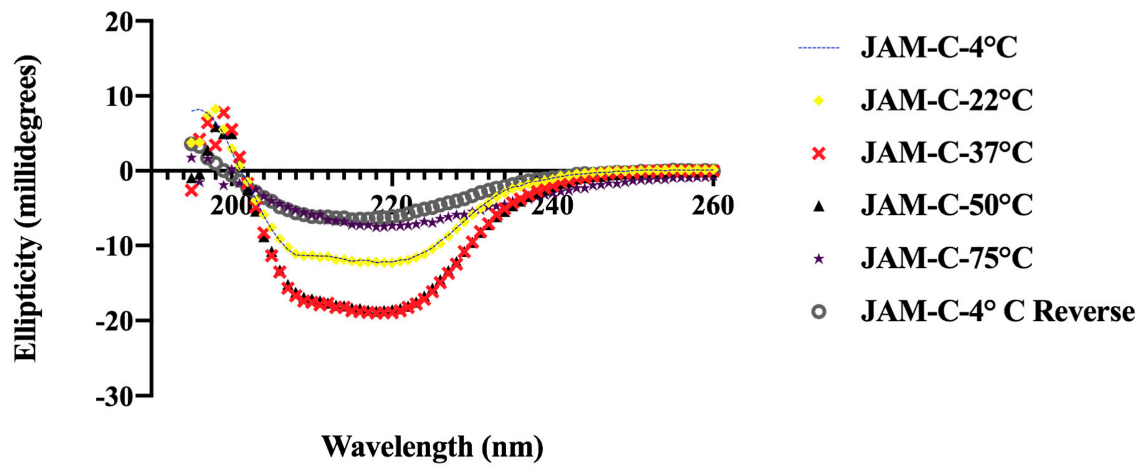


**Determining Structural Changes by Temperature of JAM 4**

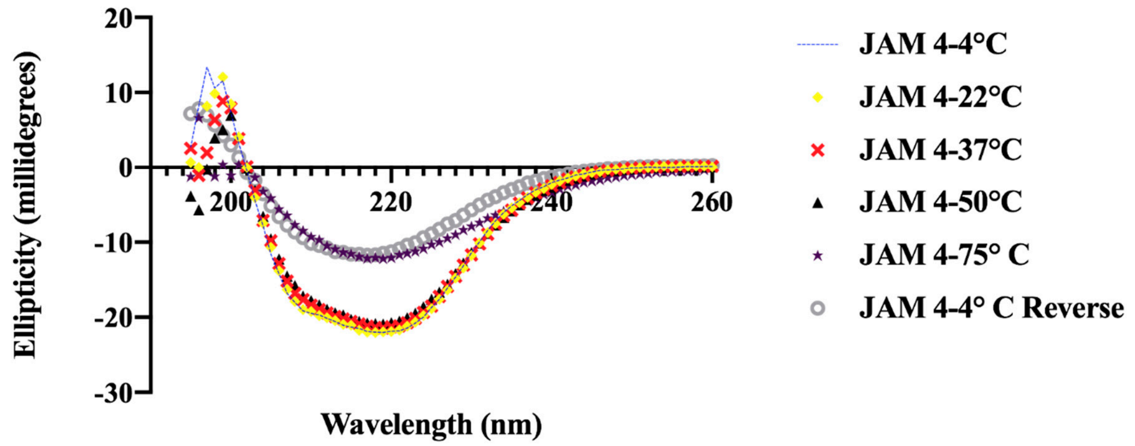

**Supplementary Table 1. Protein Yields**

| YIELD (mg/L) | |
|---|---|
| MBP | 7.2 |
| E-CAD | 1.3 |
| JAM-A | 8.6 |
| JAM-B | 0.8 |
| JAM-C | 1.9 |
| JAM4 | 1.1 |

**Supplementary Table 2. Primers for PCR amplification**

| | Forward | Reverse |
|---|---|---|
| JAM-A | atataCATATGggaagtggaagcggaag | tatataCTCGAGacggacagcattggaagtcat |
| JAM-B | tatataCATATGggaagtggaagcggaagttttagc | tatataCTCGAGacgcttaccaggacag |
| JAM-C | tatataCATATGctcgagggaagtggaagc | tatataCTCGAGttcttgttcctcgcaacgc |
| JAM4 | tatataCATATGGGCTCAGGCAGTGGCAAT | tatatactcgagGTTAACCGTAGCCGATTTG |
| E-CAD | T7 promoter | T7 Reverse |

**Funding** All funding was derived from Start-up package for Dario Mizrachi’s faculty hire, Brigham Young University.

**Author Contributions:**

**Mendoza C.** Experimental design, experiment performance, data analysis, manuscript writing. This author contributed to every aspect of the research and manuscript preparation. Conceptualization, methodology, *in silico* analysis, formal analysis, data curation and Original Draft Preparation with its accompanying review and editing. This author mentored and supervised Mr. Nagidi.

**Nagidi S.** Experimental performance of Surface Plasmon Resonance (SPR), data analysis. Aided in sample preparation

**Mizrachi D.** Experimental design, data analysis, manuscript writing. This author also contributed to manuscript preparation. Conceptualization, methodology, formal analysis, data curation and Original Draft Preparation with its accompanying review and editing. This author mentored all other co-authors.

**Conflicts of Interest**:  "The authors declare no conflict of interest."

**Original Images** All images are original and have not been altered. This is the case of Figure 1.
